# Regulation of medical diagnostics and medical devices in the East African community partner states

**DOI:** 10.1186/s12913-014-0524-2

**Published:** 2014-10-31

**Authors:** Simon Peter Rugera, Ruth McNerney, Albert K Poon, Gladys Akimana, Rehema Forgen Mariki, Henry Kajumbula, Elizabeth Kamau, Servilien Mpawenimana, Said Yusuf Said, Anthony Toroitich, Wesley Ronoh, Kimberly A Sollis, Stanley Sonoiya, Rosanna W Peeling

**Affiliations:** Department of Medical Laboratory Sciences, Mbarara University of Science and Technology, Mbarara, Uganda; Department of Clinical Research, London School of Hygiene & Tropical Medicine, London, UK; Ministry of Health, Kigali, Rwanda; Tanzania Food and Drugs Authority, Dar es Salaam, Tanzania; Central Public Laboratory, Ministry of Health, Kampala, Uganda; Makerere University College of Health Sciences, Kampala, Uganda; Kenya Medical Laboratory, Technicians and Technologists Board, Nairobi, Kenya; Department of Pharmacy and Laboratories, Ministry of Health, Bujumbura, Burundi; Zanzibar Food and Drug Board, Zanzibar, United Republic of Tanzania; Directorate of Product Evaluation and Registration, Pharmacy and Poisons Board, Nairobi, Kenya; EAC-GIZ Programme on Regional Integration, Deutsche Gesellschaft fur Internationale Zusammenarbeit, Arusha, Tanzania; East African Community Secretariat, Arusha, Tanzania

**Keywords:** Regulation, IVD, Diagnostic test, Medical device, East African community

## Abstract

**Background:**

Medical devices and *in vitro* diagnostic tests (IVD) are vital components of health delivery systems but access to these important tools is often limited in Africa. The regulation of health commodities by National Regulatory Authorities is intended to ensure their safety and quality whilst ensuring timely access to beneficial new products. Streamlining and harmonizing regulatory processes may reduce delays and unnecessary expense and improve access to new products. Whereas pharmaceutical products are widely regulated less attention has been placed on the regulation of other health products. A study was undertaken to assess regulation of medical diagnostics and medical devices across Partner States of the East African Community (EAC).

**Methods:**

Data was collected during October 2012 through desk based review of documents and field research, including face to face interviews with the assistance of a structured questionnaire with closed and open ended questions. Key areas addressed were (i) existence and role of National Regulatory Authorities; (ii) policy and legal framework for regulation; (iii) premarket control; (iv) marketing controls; (v) post-marketing control and vigilance; (vi) country capacity for regulation; (vii) country capacity for evaluation studies for IVD and (viii) priorities and capacity building for harmonization in EAC Partner States.

**Results:**

Control of medical devices and IVDs in EAC Partner States is largely confined to national disease programmes such as tuberculosis, HIV and malaria. National Regulatory Authorities for pharmaceutical products do not have the capacity to regulate medical devices and in some countries laboratory based organisations are mandated to ensure quality of products used. Some activities to evaluate IVDs are performed in research laboratories but post market surveillance is rare. Training in key areas is considered essential to strengthening regulatory capacity for IVDs and other medical devices.

**Conclusions:**

Regulation of medical devices and *in vitro* diagnostics has been neglected in EAC Partner States. Regulation is weak across the region, and although the majority of States have a legal mandate to regulate medical devices there is limited capacity to do so. Streamlining regulation in the EAC is seen as a positive aspiration with diagnostic tests considered a priority area for harmonisation.

**Electronic supplementary material:**

The online version of this article (doi:10.1186/s12913-014-0524-2) contains supplementary material, which is available to authorized users.

## Background

The East African Community (EAC) is a regional inter-governmental organization of five Partner States, namely the Republic of Kenya, the Republic of Uganda, the Republic of Burundi, the Republic of Rwanda and the United Republic of Tanzania. Together the five states cover an area of approximately 1.8 million square kilometres and a population of over 135 million people. In addition to promoting trade and economic growth a priority consideration for the EAC is the health of the population. Health indicators across the region show increasing life expectancies [1] but despite progress made the region remains beset by conditions such as tuberculosis, HIV/AIDS, other sexually transmitted infections and in some geographic areas malaria [2–4]. The 1999 founding Treaty for the Establishment of the East African Community articulates a policy to “harmonize national health policies and regulations and promoting the exchange of information on health issues in order to achieve quality health within the Community” [5]. A program to harmonize registration of medicines within EAC Partner States was launched in 2012 under the leadership of the EAC Medicines and Food Safety Unit [6]. The project forms part of a wider programme, the African Medicines Regulatory Harmonisation (AMRH) Initiative which is coordinated by the African Union’s New Partnership for Africa’s Development (NEPAD). The overall aim of the AMRH Programme is to improve public health in Africa by increasing access to good quality, safe and effective medicines through the harmonization of medicines regulations, including reduction of the time taken to register essential medicines for the treatment of diseases [6].

In addition to medicines other medical products of benefit to patients include medical devices and diagnostic tests. Such products make a major contribution to health as many important diseases and conditions require diagnostic guidance to ensure appropriate care. This includes therapeutic use of drugs, where delayed diagnosis can result in deterioration of the patient and, for infectious conditions, continued transmission and spread of disease. The regulation of products for human health by National Regulatory Authorities (NRAs) is intended to ensure their safety and quality whilst ensuring timely access to beneficial new products. Safety may be assured by pre-market scrutiny of new products and by post- market surveillance to ensure quality and performance are maintained. Regulatory controls imposed should be proportionate to the risk of harm to individual or public health. The market dynamics for medical devices and diagnostic products for health are distinct from those for pharmaceutical products and their regulation requires a different approach to that for medicines. Medical devices present a wide range of technologies, ranging from the simple band aid to very complex machinery. Quality of design and manufacture is important, particularly for devices that use electrical power where a malfunction could cause serious injury or death or for products that use toxic reagents or expose the user to risk of infection.

Diagnostic tests vary in complexity, ranging from simple colorimetric dip-stick devices to sophisticated computerised instruments and for some conditions there is a proliferation of products suitable for use at different levels of the healthcare delivery system. Most diagnostic tests fall into the category of *in vitro* diagnostics (IVDs), where they are used to test specimens obtained from the patient. Unlike medicines or vaccines the product is not ingested and the risk of causing harm is related to the performance of the device and likelihood of a malfunction and the consequences of obtaining an incorrect test result. The Global Harmonization Task Force have recommended a four tier risk classification system for medical devices and diagnostics where risk is defined as the combination of the probability of occurrence of harm and the severity of that harm [7].

Reagents such as microbiological stains or culture media fall into the lowest category as they pose little hazard whereas for tests used to screen blood products for deadly diseases such as HIV an incorrect result could have serious consequences, including onward transmission of the disease. Such high risk products require more stringent regulation, including studies of clinical performance to determine their efficacy in the target population.

Access to IVDs and other medical devices in developing countries is currently limited by their availability and cost and in addition many diagnostic tests require laboratory facilities and trained personnel that are not readily available in Africa. A new generation of devices are being developed for diagnosing important diseases that promise significant benefit to public health. These include tests for use at point-of-care that do not require referral to specialist facilities [8]. It is important that patients in developing countries have timely access to these products and that they are made available at affordable prices. Regulation of medical devices in developing countries is weak and this is particularly a problem with diagnostic tests [9]. In countries that do not regulate IVDs, they may be sold with little or no evidence of their efficacy. Where controls do occur they can act as a barrier to products entering the country [10]. An example is the delayed marketing of a point-of-care device for enumerating CD4 cells to enable patients with HIV to access therapy. The requirement for clinical trials in individual countries has resulted in considerable duplication of effort with little or no scientific gain, and has increased costs and delayed their introduction in some African countries by years.

In accordance with EAC policy to improve public health through harmonized regulation, a regional survey was undertaken during 2012 of the regulation of medical diagnostics and medical devices in the East African Community Partner States. The aim of the survey was to assess capacity and current practices across the Partner States for regulation of medical diagnostics (including *In-vitro* Diagnostics) and medical devices and the capacity to undertake pre and post market assessment of quality, safety and effectiveness. We hereby report the findings of the study and recommendations made following review of the data by a panel of technical experts drawn from EAC partner states.

## Methods

Data was collected during October 2012 through desk based review and field research, including face to face interviews. Information was collected with the assistance of a structured questionnaire containing both closed and open ended questions [see Additional file 1: Appendix S2 for detailed questionnaire]. Questions were framed to collect data on each of the essential features of a regulatory program for medical devices as described in guidelines published by the World Health Organisation [11]. The questionnaire addressed *in vitro* diagnostic devices and medical devices using the following definitions adapted from guidance issued by the Global Harmonization Task Force, a voluntary group of representatives from medical device regulatory authorities and trade associations from Europe, the United States of America (USA), Canada, Japan and Australia [12].

*A medical device (MD) is an instrument, apparatus, implant, in vitro reagent, or other similar or related article, which is intended for use in the diagnosis of disease or other conditions, or in the cure, mitigation, treatment, or prevention of disease, or intended to affect the structure or any function of the body and which does not achieve any of its primary intended purposes through chemical action within or on the body*.

*An ‘in vitro diagnostic device’ (IVD) is a medical device which is a reagent, reagent product, calibrator, control material, kit, instrument, apparatus, equipment, or system, whether used alone or in combination, intended by the manufacturer to be used in vitro for the examination of specimens, including blood and tissue donations, derived from the human body, solely or principally for the purpose of providing information concerning a physiological or pathological state.*

Key areas addressed were (i) existence and role of National Regulatory Authorities; (ii) policy and legal framework for regulation; (iii) premarket control; (iv) marketing controls; (v) post-marketing control and vigilance; (vi) country capacity for regulation; (vii) country capacity for evaluation studies for IVD and (viii) priorities and capacity building for harmonization in EAC Member States.

Each Partner State was visited to enable interview with key stakeholders. Interviewees were selected on the recommendation of country experts. Both mainland Tanzania and the semi- autonomous territory of Zanzibar were consulted. Where experts were not available for interview information was sourced from documented evidence. In addition to the completed questionnaires written reports were received from country experts. Information sources used in the study are summarised in Table 1 and a list of documents accessed is available in the Additional file 2: Table S1.

**Table 1:** **Summary of evidence sources**

**Country**	**Documents reviewed**	**Organisations interviewed (Number of persons)**	
Burundi	1	i.	Ministry of Health
		ii.	Department of Pharmacy, Medicines and Laboratory
Kenya	17	i.	Pharmacy and Poisons Board (3)
		ii.	National Quality Control and Medical Devices Laboratory
		iii.	Kenya Medical Laboratory Technicians and Technologists Board (2)
Rwanda	4		No organisations interviewed
Tanzania (Mainland)	11	i.	Tanzania Food and Drugs Authority (2)
		ii.	Private Health Laboratories Board
Tanzania (Zanzibar)	6	i.	Zanzibar Food and Drugs Board (2)
		ii.	Central Medical Stores, Ministry of Health and Social Welfare
		iii.	Chief Pharmacist, Ministry of Health and Social Welfare
Uganda	8	i.	National Drug Authority (3)
		ii.	Pharmacy Division, Ministry of Health
		iii.	Uganda National Bureau of Standards (2)
		iv.	Allied Health Professionals Council
		v.	Medilab (Laboratory supplies company)
		vi.	Central Public Health Laboratories
Total	47	16	(24)

Following compilation, the data was reviewed by a panel of experts from the Republic of Kenya, the Republic of Uganda, the Republic of Burundi and the United Republic of Tanzania (Mainland and Zanzibar), including representatives of Ministries of Health, National Regulatory Authorities, National Medical Laboratories and Public Health Regulatory Authorities. Representatives of medical diagnostics manufacturers were also present.

Ethical review was not applicable to this study which accessed and collated information in the public domain. No ‘study subjects’ participated in the study, no human data or materials were collected and no interventions were performed. All persons interviewed agreed to do so by prior appointment as part of their professional attachment to the institutions. This study was undertaken at the request of the Council of Ministers of Health of the East African Community partner states.

## Results and discussion

Reports were compiled from all five partner States. A total of 24 interviews were conducted representing 16 organisations and 47 documents were collated (Table 1).

### Legislative and policy framework

Four Member States, Burundi, Kenya, Rwanda and Tanzania (Mainland and Zanzibar) reported legislation in the form of Acts of Parliament addressing products for health, including medical diagnostics and medical devices (Table 2). Both Kenya and Tanzania Mainland reported legislative inconsistencies relating to medical devices used in laboratories, with some duel responsibility for their control. The fifth Partner State, Uganda has legislation regarding the control of drugs (National Drug Policy and Authority Act 1993) but no provision for the regulation of medical products other than general legislation for all commodities which are defined as ‘any article, product or thing which is or will ultimately be the subject of trade or use’. (The Uganda National Bureau Of Standards Act 1983).

**Table 2:** **Regulatory legislature and health policy in EAC Member States and territories**

	**Legislation for medical devices**	**National Health Policy**	**National Health Laboratory Policy**	**Guidelines**	**National Regulatory Authority**
Burundi	Yes	na	na	na	Directorate of Pharmacies, Medicines and Laboratories
Kenya	Yes	Yes	Yes	Yes	Kenya Medical Laboratory Technicians and Technologist Board/Pharmacy and Poisons Board
Rwanda	Yes	Yes	na	Yes	Task force to set up FDA, but diagnostics not yet included
Tanzania Mainland	Yes	Yes	P	Yes	Tanzania Food and Drug Authority/Private Health Lab Board
Tanzania Zanzibar	Yes	Yes	na	Yes	Zanzibar Food and Drugs Board
Uganda			Yes	Yes	National Drug Authority/Allied Health Professionals Council of Uganda

*na*: Not available; *P*: Aspects of laboratory policy incorporated in the National Health Policy.

However, guidelines on use of medical diagnostic products are being developed by the Allied Health Professionals Council (AHPC) of Uganda. The AHPC is mandated by an Act of Parliament to regulate medical laboratory professionals (among other Allied Health Professional workers) and any other matter related to the practice of the professionals.

All EAC Partner States identified one or more regulatory bodies for medical products with the exception of Rwanda that has instigated a taskforce to oversee the establishment of a Food and Drug Authority. Whereas in all States medicines are regulated by either a Pharmacy Board or a Food and Drugs Authority there is little capacity for regulation of medical devices or IVDs. Where medical devices are controlled it is largely within disease specific programmes such as tuberculosis, malaria or HIV/AIDS with the Department of Health or international donors guiding procurement decisions rather than guidance from a national regulatory authority. Two lists of approved products were supplied by respondents to the questionnaire. The Private Health Laboratories Board in Tanzania list included IVDs for HIV, malaria, hepatitis and syphilis and a number of Haematology and Chemistry Analysers. The Kenyan National AIDS and STI Control Programme (NASCOP) provided a list of diagnostic products approved for HIV, hepatitis B and C and syphilis. One country, Tanzania, reported that public procurement was linked to regulation through consultation with the Private Health Laboratories Board. Both Burundi and Uganda require authorization of importation.

Some countries reported dual responsibility for diagnostic devices where more than one agency is mandated and some tensions were evident between laboratory-based organizations who evaluate diagnostic test performance and agencies whose primary activities lie in the regulation of medicines. Although the focus of regulatory activities was pharmaceuticals some countries are taking steps towards strengthening diagnostics regulation, including Tanzania who are receiving support through a project with the World Health Organisation. New guidelines are under consideration and are expected to result in some realignment of activities with the Tanzanian Food and Drug Authority assuming some duties currently under the remit of the Private Health Laboratories Board. Policies in Kenya are similarly under review with the aim to better define the respective roles of the Kenya Medical Laboratory Technicians and Technologist Board (KMLTTB) which has responsibility for the quality of medical laboratory activities and the Pharmacy and Poisons Board which regulates medicines.

### Pre-market regulation

Whereas some regulatory activity was reported in each Partner State more detailed questioning revealed that with the exception of Tanzania pre market regulation of medical devices and IVDs by National Regulatory Authorities is largely absent across the EAC (Table 3). No audit visits to manufactures to assess quality systems are currently undertaken by EAC Partner States. There is some scrutiny of products for the control of TB, malaria, HIV and other STIs and while some States accept products approved by donor agencies without further testing others choose to undertake pre market testing of IVDs in local laboratories.

**Table 3:** **Control of diagnostic and medical devices in EAC Partner States**

**Country**	**Burundi**	**Kenya**	**Rwanda**	**Tanzania Mainland**	**Tanzania Zanzibar**	**Uganda**
Are medical devices regulated by a National Authority	Some	Some	Programme only (MOH)	Yes	Joint with Mainland (TFDA)	Yes
Regulation of in-vitro diagnostics	Not yet	In development	Programme only (MOH)	Yes	Joint with TFDA	Guidelines (Allied Health Professionals Council of Uganda)
Regulation of IVDs in the private sector	Not yet	In development	N/A	Yes	Joint with TFDA	Guidelines (Allied Health Professionals Council of Uganda)
Regulation lab reagents and stains for diagnostics use	Import controls	In development	N/A	Yes	Joint with TFDA	Guidelines (Allied Health Professionals Council of Uganda)
Regulation of diagnostic tests for veterinary use	None	In development	None	Yes	Joint with TFDA	Yes
Pre-market control on IVDs	None	None	Programme only^1^	Yes	Joint with TFDA	None
Post market control on IVDs	None	None	Programme only^1^	Yes	Joint with TFDA	None

^1^Control programmes supported by donor aid e.g. TB, HIV/AIDS, Malaria.

Some harmonization of regulatory processes was observed with cooperation between the Food and Drug Authorities of Tanzania mainland and Zanzibar with mutual recognition of some activities. Tanzania also reported that for products with prior regulatory approval from a stringent National Regulatory Authority (Canada, Japan, Australia, EU and USFDA) or prequalification by WHO could be reviewed using an abridged dossier.

### Market and post market controls

In the absence of regulatory capacity for medical devices and IVDs market controls in EAC Partner States are not specific for these products. Three States, Burundi, Kenya and Tanzania require manufacturers to have a local agent with legal accreditation prior to registering a product for distribution in the country. Authorization for import is required in Tanzania and Uganda, with some enforcement in Tanzania through warehouse inspections.

Advertising controls were reported in Burundi, Tanzania and Uganda with some vetting and pre approval required. Post market regulation was reported as being reactive rather than proactive. Tanzania and Uganda reported that reactive investigations were undertaken if problems were reported. In Rwanda post market monitoring is undertaken by Programmes (HIV/AIDS, TB etc.). The Private Health Laboratories Board in Tanzania was the only organization that reported a mechanism for tracking medical devices or guidelines for recalling substandard medical devices or IVDs, although some other States reported mechanisms for reporting and recall of pharmaceuticals.

### National capacity

Data supplied by Member States suggests that capacity to regulate either medical devices or diagnostic tests within the EAC is limited. Capacity is divided between persons with laboratory based technical expertise regarding the use of medical devices and persons with regulatory experience of pharmaceuticals but little or no knowledge of IVD or other medical devices. Regulation of some IVD that are considered high risk to individual or public health requires knowledge of their efficacy obtained from clinical trials to assess their performance in the target population. Little direct involvement of regulatory agencies in IVD clinical trials was reported but all countries reported some capacity for laboratory assessment of IVD. In addition Burundi, Kenya, Tanzania (mainland) and Uganda reported capacity for clinical trials for devices applicable to TB, HIV and malaria. Formalized Technology Assessment Programmes are absent in all EAC Partner States but both Uganda and Kenya have research institutions with some experience in technology assessment for IVD.

The quality of laboratory studies is critical for both pre market evaluation and post market surveillance and accreditation of laboratories is a highly desirable prerequisite. Respondents from Kenya and Uganda reported a number of laboratories accredited to international standards and Tanzania is currently engaged in a programme designed to lead to laboratory accreditation.

### Priorities

When asked to state priorities for harmonization the most common response across respondents was the need to harmonize regulation of rapid diagnostic tests for important diseases, with malaria, TB and HIV given as examples. The need to strengthen existing National Regulatory Authorities was also highlighted. Suggestions for future harmonization activities included use of a common nomenclature and definitions, mutual recognition and reducing the number of clinical trials. Priorities for capacity building most frequently included training to improve and maintain the knowledge base and skills of personnel. Areas where support was requested included dossier evaluation and review, development of protocols and Standard Operating Procedures, quality management systems and post market monitoring and surveillance. Some respondents recognised the need to differentiate training dependant on role, recognising expertise in clinical trials or review of submission dossiers.

### Recommendations

Following review of the data and the expert meeting of stakeholders from EAC Partner States, a series of general recommendations were made as listed below. The recommendations were made in the context of opportunities for harmonizing regulation to accelerate access to affordable and safe new products. Topics covered included the legal framework, product nomenclature and classification and mechanisms of harmonizing regulation including formation of technical working groups and task forces, communication platform within the region and use of global guidelines and relationship to other working harmonization parties.

### List of recommendations arising from stakeholders meeting

- Diagnostic products should be considered a priority for regulatory harmonisation in EAC Partner States and there should be an extension plan for the regulatory framework to cover medical devices in the longer term.
- Each Partner State should have a legal and policy framework for medical diagnostics and devices suitable for harmonisation. The EAC should collaborate with EAC partner states to expedite IVDs and medical devices legislation and policy framework where it does not exist; and to identify refinement areas to existing ones where it is already in the law system.
- Common nomenclature and product classifications should be used across the EAC
- Partner States should develop mechanisms and capacity for regulation that would feed into EAC harmonised regulation
- EAC and Partner States should establish a communication platform to enable the necessary exchange of information relating to medical diagnostics and medical devices in order to provide prompt safety information for the nations to safeguard patients.
- There is need for donor support with regard to medical diagnostics for:
  Facilitating appropriate legal frame work in each partner states as a matter of urgency
  Capacity building and training needs support for NRA;
  Facilitating harmonisation in the EAC partner states
  Facilitating formation of a Pan African Harmonisation Working Party (PAHWP)
- Technical Working Groups should be established to pursue harmonization activities.
- An EAC Regional Task Force on Harmonization and Regulation of Medical Diagnostics and Medical Devices should be established
- The task force should lead to the establishment of a Pan-African Harmonization Working Party (PAHWP)
- Harmonization activities should be undertaken with consideration to guidelines issued by the Global Harmonization Task Force and activities of other regional bodies such as the Asian Harmonization Working Party.

## Conclusions

This study was undertaken to inform the EAC Secretariat and EAC Partner States of the current status of regulatory activities for medical devices and medical diagnostics within the EAC region. Data and opinions collected indicate that regulation of medical devices and *in vitro* diagnostics is a neglected area. Regulation is weak across the region, and although the majority of Member States have a legal mandate to regulate medical devices there is limited capacity to do so. Responses received suggest that harmonization and streamlining regulation in the EAC is seen as a positive aspiration. That rapid IVD to detect important infectious diseases are seen as a priority for harmonisation is not surprising in light of health needs in this region of Africa. However, a limitation of the study is that due to restraints of time and finance the list of stakeholders consulted was not exhaustive and it is possible that those who were not available for comment would be less enthusiastic about the regulation of IVD and medical devices. Several respondents indicated that policy reviews are being undertaken and at least one State (Tanzania) is proposing changes to the current regulatory framework and process. The collaboration between the NRAs in United Republic of Tanzania is fertile ground for regulatory harmonisation process in the region. Future activities planned include establishing a Task Force and Technical Working Groups to take forward strengthening and streamlining regulatory activities in the EAC and it is clear that investment shall be needed to increase capacity.

## Additional files

Additional file 1:
**Appendix S2 - Questionnaire.**
Additional file 2: Table S1. Documents Accessed.
