# Supplementary material for: Regulation of medical diagnostics and medical devices in the East African community partner states
Source: BMC Health Serv Res. 2014 Oct 31;14:524. doi: 10.1186/s12913-014-0524-2 (PMC4221680; doi:10.1186/s12913-014-0524-2)
Supplement: Additional file 1: — Appendix S2 - Questionnaire. [file 12913_2014_524_MOESM1_ESM.doc]

Appendix S2

**Questionnaire**

**REGIONAL BASELINE SURVEY ON THE REGULATION OF MEDICAL DIAGNOSTICS AND DEVICES IN THE EAST AFRICAN COMMUNITY PARTNER STATES**

The purpose of this survey is to capture the current status regarding the regulation of medical diagnostics (including *In-vitro* Diagnostics) and medical devices within the East African Community and assess capacity to undertake pre and post market assessment of the quality, safety and efficacy of these devices.

The survey has been commissioned with financial assistance from GIZ and Grand Challenges Canada following a two-day workshop on harmonization of regulation of in vitro diagnostics held by The East African Community Secretariat in collaboration with the EAC Partner States’ National Medical and Public Health Laboratory Regulatory Authorities, the African Union/NEPAD Planning and Coordination Agency, the London School of Hygiene & Tropical Medicine (LSHTM), GIZ and other international cooperating partners at the Kenya Medical Research Institute (KEMRI) headquarters in Nairobi, Kenya from 30th to 31st July 2012.

For the purposes of this survey the following definitions shall apply:

A **medical device (MD)** is an instrument, apparatus, implant, in vitro reagent, or other similar or related article, which is intended for use in the diagnosis of disease or other conditions, or in the cure, mitigation, treatment, or prevention of disease, or intended to affect the structure or any function of the body and which does not achieve any of its primary intended purposes through chemical action within or on the body.

An ‘**in vitro diagnostic device’ (IVD)** is a medical device which is a reagent, reagent product, calibrator, control material, kit, instrument, apparatus, equipment, or system, whether used alone or in combination, intended by the manufacturer to be used in vitro for the examination of specimens, including blood and tissue donations, derived from the human body, solely or principally for the purpose of providing information concerning a physiological or pathological state.

**Completed questionnaires must be returned by XX^th^ October.**

**Please return to XXXXX Email: XXXX**

**Please complete each section of the form as fully as possible, using additional sheets where necessary. Where you are not able to provide a response please indicate by entering N/A.**

**SECTION A National Regulatory Authorities**

| **1.** | **Country** |  |
| --- | --- | --- |

| **2.** | **National Regulatory Authority(ies) (NRA)** |  |
| --- | --- | --- |

| **3.** | **Are medical devices regulated by your NRA?** |  |
| --- | --- | --- |

| **4.** | **Which types of medical devices are regulated by the NRA (if any)?** |  |
| --- | --- | --- |

| **5.** | **Which medical devices for diagnostic use (excluding IVD) are regulated by the NRA? (if any)** |  |
| --- | --- | --- |

| **6.** | **Are medical devices used in the private sector regulated by the NRA?** |  |
| --- | --- | --- |

| **7.** | **Are medical devices from donor programmes regulated by your NRA?** |  |
| --- | --- | --- |

| **8.** | **Are in vitro diagnostics (IVD) for blood products regulated by your NRA?** |  |
| --- | --- | --- |

| **9.** | **Are in vitro diagnostics (IVD) not for blood products regulated by your NRA?** |  |
| --- | --- | --- |

| **10.** | **Are in vitro diagnostics (IVD) from donor programmes regulated by your NRA?** |  |
| --- | --- | --- |

| **11.** | **Are IVD used in the private sector regulated by the NRA?** |  |
| --- | --- | --- |

| **12.** | **Are laboratory reagents and stains for diagnostic use regulated?** |  |
| --- | --- | --- |

| **13.** | **Is there a mandate for the NRA to regulate diagnostic tests for veterinary use** |  |
| --- | --- | --- |

**SECTION B Regulation of in vitro diagnostics**

| **14.** | **Legal framework for the regulation of IVD** | *Please provide name/date of relevant acts and a brief summary.* |
| --- | --- | --- |
| **a.** | Pre market registration |  |
| **b.** | Manufacturing controls |  |
| **c.** | Import controls relating to IVD |  |
| **d.** | Export controls relating to IVD |  |
| **e.** | Marketing controls (advertising, labeling etc) |  |
| **f.** | Post-market controls (post market vigilance and surveillance, adverse incident reports etc) |  |
| **g.** | What enforcement agencies are used (if any). |  |
| **h.** | Is the legistlation enforceable in both the public and private healthcare sectors. |  |

| **15.** | **Premarket controls for IVD** |  |
| --- | --- | --- |
| a. | Risk Based classification system |  |
| b. | Premarket evaluation – document based. Summary technical Document (STED) |  |
| c. | Premarket evaluation –laboratory testing/clinical trials. |  |
| d. | Manufacturing site quality management system |  |
| e. | Manufacturing site inspection & quality audits |  |
| f. | Does prior approval from an external stringent NRA such as US FDA  expediate approval |  |

| **16.** | **Marketing Controls for IVD** |  |
| --- | --- | --- |
| a. | Advertising control |  |
| b. | Labelling and instructions for use control |  |
| c. | Is procument within the public sector linked to product registration? |  |
| d. | Is procument within the private sector linked to product registration? |  |

| **17.** | **Post-marketing Controls for IVD** |  |
| --- | --- | --- |
| a. | Post approval studies |  |
| b. | Device tracking |  |
| c. | Device reporting |  |
| d. | Mis branding and adulteration |  |
| e. | Corrections/Recall |  |

| **18.** | **Accessibility** |  |
| --- | --- | --- |
| a. | Does the NRA have a website for IVD registration? |  |
| b. | Can registration forms be downloaded |  |
| c. | Is a list of fees available |  |
| d. | Is an ‘in country’ agent (local responsible party) a pre requirement for product registration. |  |
| e. | How long is the current registration process for IVD. |  |

| **19.** | **Which IVD currently have NRA approval?** |  |  |
| --- | --- | --- | --- |

| **20.** | **Which IVD not assessed/regulated by the NRA have MOH approval?** |  |
| --- | --- | --- |

| **21.** | **NRA Capacity** |  |
| --- | --- | --- |
| a. | Pre-market for IVD |  |
| b. | Marketing controls for IVD |  |
| c. | Post market controls for IVD |  |

| **22.** | **Capacity for evaluation studies for IVD** |  |
| --- | --- | --- |
| a. | Clinical trial design |  |
| b. | Laboratory capacity |  |
| c. | Laboratory acredition |  |
| d. | Technology assessment |  |

**SECTION C Regulation of medical devices**

| **23.** | **Legal framework for the regulation of medical devices** |  |
| --- | --- | --- |
| **a.** | Pre market registration |  |
| **b.** | Manufacturing controls |  |
| **c.** | Import controls relating to MD |  |
| **d.** | Export controls relating to medical devices |  |
| **e.** | Marketing controls (advertising, labeling etc) |  |
| **f.** | Post-market controls (post market vigilance and surveillance, adverse incident reports etc) |  |
| **g.** | What enforcement agencies are used (if any). |  |
| **h.** | Is the legistlation enforceable in both the public and private healthcare sectors. |  |

| **24.** | **Premarket controls for medical devices** |  |
| --- | --- | --- |
| a. | Risk Based classification system |  |
| b. | Premarket evaluation – document based (Common Submission Dossier Template) |  |
| c. | Documented safety, quality and efficacy |  |
| d. | Manufacturing site quality management system |  |
| e. | Manufacturing site inspection & quality audits |  |
| f. | Accreditation of conformity assessment bodies (CAB) and notified bodies (NB) |  |

| **25.** | **Marketing Controls for medical devices** | *Please describe system used* |
| --- | --- | --- |
| a. | Advertising control |  |
| b. | Labelling and instructions for use control |  |
| c. | Good distribution practice |  |
| d. | Is procument in the public sector linked to product registration |  |
| e. | Is procument in the private sector linked to product registration |  |

| **26.** | **Post-marketing Controls for medical devices** | *Please describe system used* |
| --- | --- | --- |
| a. | Device tracking |  |
| b. | Device reporting |  |
| c. | Complaints handling |  |
| d. | Corrections/Recall |  |

| **27.** | **Accessibility** |  |
| --- | --- | --- |
| a. | Does the NRA have a website |  |
| b. | Can registration forms be downloaded |  |
| c. | Is a list of fees available |  |
| d. | Is an ‘in country’ agent (local responsible party) a pre requirement for product registration. |  |
| e. | How long is the current registration process for medical devices. |  |

| **28.** | **NRA Capacity** |  |
| --- | --- | --- |
| a. | Pre-market |  |
| b. | Marketing controls for medical devices |  |
| c. | Post market controls for medical devices |  |

**SECTION D. Priorities and capacity building for harmonization of diagnostic devices in EAC Member States.**

| **29.** | **What is your recommended priority for harmonized regulatory control of diagnostic devices?** |  |
| --- | --- | --- |

| **30.** | **What is your recommended priority for harmonized regulatory activities for diagnostic devices?** |  |
| --- | --- | --- |

| **31**. | **Capacity building/training needs.** |  |
| --- | --- | --- |

| **32.** | **Any additional comments** |  |
| --- | --- | --- |
