# Supplementary material for: Regulation of medical diagnostics and medical devices in the East African community partner states
Source: BMC Health Serv Res. 2014 Oct 31;14:524. doi: 10.1186/s12913-014-0524-2 (PMC4221680; doi:10.1186/s12913-014-0524-2)
Supplement: Additional file 2: Table S1. — Documents Accessed. [file 12913_2014_524_MOESM2_ESM.doc]

Supplementary materials

Regulation of Medical Diagnostics and Medical Devices in the East African

Community Partner States. Simon Peter Rugera *et al*

Table S1 Documents Accessed

|  | Document title | Publisher |
| --- | --- | --- |
| B1 | Burundi baseline survey report | Servilien Mpawenimana  (Burundi) |
| K1 | Kenyan Health Policy 2012-2030 | Ministry of Medical Services and  Ministry of Public Health and  Sanitation (Kenya) |
| K2 | Medical Laboratory Services of Kenya National Policy  Guidelines 2006 | Ministry of Health (Kenya) |
| K3 | Regulation Of In-Vitro Devices | Medical Laboratory Board  (Kenya) |
| K4 | Pharmacy and Poisons Board National Report for The  Regulation of Medical Diagnostics (Including IVDs) and  Medical Devices | Pharmacy and Poisons Board  Kenya |
| K5 | List of Approved Test Kits | Ministry of Health National AIDs  & STI Control Programme- NASCOP (Kenya) |
| K6 | Guidelines on submission of Documentation for  Registration of Medical Devices 1st Edition (2011) | Pharmacy and Poisons Board  Kenya |
| K7 | National Policy Guidelines for  Medical Laboratory Equipment Management (2012) | Ministry of Health (Kenya) |
| K8 | National Quality Management Guidance Framework for  HIV Testing and Counselling in Kenya | Ministry of Health-NASCOP  (Kenya) |
| K9 | National Guidelines for HIV Testing and Counselling in  Kenya. 2nd Edition 2010 | National AIDS and STI Control  Programme (NASCOP) Ministry of Public Health and Sanitation (Kenya) |
| K10 | Sessional Paper on National Pharmaceutical  Policy (2010) | Ministry of Medical Services  & Ministry of Public Health and  Sanitation (Kenya) |
| K11 | Draft National Policy Guidelines on Parasitological  Diagnosis of Malaria and Malaria Vector Surveillance in  Kenya | Ministry of Public Health and  Sanitation (Kenya) |
| K12 | The Pharmacy and Poisons Act. Chapter 244  Revised Edition 2009 | National Council for Law  Reporting (Kenya) |
| K13 | The Constitution of Kenya, 2010. | National Council for Law  Reporting (Kenya) |
| K14 | The Standards Act | National Council for Law  Reporting (Kenya) |

| K15 | The Medical Laboratory Technicians and  Technologists Act. Kenya Subsidiary Legislation, 2011 | Kenya Medical Laboratory  Technicians & Technologists  Board (Kenya) |
| --- | --- | --- |
| K16 | The Kenya Food and Drugs Administration Bill 2012  (Draft) | Ministry of Health (Kenya) |
| K17 | Kenya Medical Laboratory Technicians & Technologists  Board Strategic Plan 2012-2017 | Kenya Medical Laboratory  Technicians & Technologists  Board (Kenya) |
| R1 | Law N°12/99 Relating To The Pharmaceutical Art (1999) | Ministry of Justice |
| R2 | Assessment of Medicine Quality Assurance in Rwanda:  Overview of Findings and Recommendations for  Consideration (2009) | Promoting the Quality of  Medicines Program (Rwanda) |
| R3 | Draft National Pharmaceutical Policy (2009) | Ministry of Health (Rwanda) |
| R4 | Rwanda Health Financing Policy | Ministry of Health (Rwanda) |
| T1 | The Tanzania Food, Drugs and Cosmetics Act, 2003 | Government (Tanzania  mainland) |
| T2 | Draft Guidelines For Evaluation of Applications For  Registration of In Vitro Diagnostics Medical Devices  TFDA/DMC/DMDAE/G/005 | Tanzania Food and Drugs  Authority (Tanzania mainland) |
| T3 | Guidelines For Assessment of Applications For  Registration of Medical Devices | Tanzania Food and Drugs  Authority (Tanzania mainland) |
| T4 | Sera Ya Afya - National Health Policy (2007)  (Document In Swahili) | Ministry of Health and Social  Welfare (Tanzania mainland) |
| T5 | Guidance For Application of Import Permit For  Medical Devices | Tanzania Food and Drugs  Authority (Tanzania mainland) |
| T6 | Guidelines on Submission of Documentation  For Registration of Medical Devices (2009) | Tanzania Food and Drugs  Authority (Tanzania mainland) |
| T7 | Private Health Laboratories Act 1997 | Government (Tanzania mainland) |
| T8 | Proposed Amendments of The Private Health Laboratory  Regulation Act, No. 10 Of 1997  Proposed Amendments of The Tanzania Food, Drugs and  Cosmetics Act, No. 1 Of 2003 | (Tanzania mainland) |
| T9 | Notification Of In Vitro Diagnostics Form | Ministry of Health and Social  Welfare (Tanzania mainland) |
| T10 | The Standards Act 2009 | Government (Tanzania  mainland) |
| T11 | Report on Baseline Survey For Regulation of Medical  Diagnostics (Including IVDs) and Medical Devices | Rehema Mariki, Tanzania Food  and Drugs Authority (Tanzania mainland) |
| T12 | The Zanzibar Food, Drugs And Cosmetics  Act, No. 2 Of 2006 | Government of Zanzibar  Ministry of Health (Tanzania  Zanzibar) |
| T13 | Zanzibar Health Sector  Performance Report 2010/2011 | Government of Zanzibar  Ministry of Health (Tanzania  Zanzibar) |
| T14 | Guideline On Application Of Submission Of Medical  Devices (2012) | Zanzibar Food Drugs and  Cosmetics Board (Tanzania  Zanzibar) |
| T15 | National Report for the Regulation of Medical Diagnostics  (Including IVDs) and Medical Devices. | Said Yussuf Said (Tanzania  Zanzibar) |

23

| T16 | Zanzibar National Drug Policy (2006) | Ministry of Health and Social  Welfare (Tanzania Zanzibar) |
| --- | --- | --- |
| T17 | Zanzibar Medicine Policy Implementation Plan  2008 – 2012 | Ministry of Health and Social  Welfare (Tanzania Zanzibar) |
| U1 | THE UGANDA NATIONAL BUREAU OF STANDARDS ACT  1983 | Uganda Legal Information  Institute (Uganda) |
| U2 | Uganda National Health Laboratory  Services Policy (2009) | Ministry of Health (Uganda) |
| U3 | The Allied Health Professionals Act (1996) | Uganda Legal Information  Institute (Uganda) |
| U4 | National Drug Policy and Authority Act 1993 | Uganda Legal Information  Institute (Uganda) |
| U5 | National Drug Authority Guidelines for the conduct of  clinical trials | National Drug Authority,  Ministry Of Health (Uganda) |
| U6 | Guidelines On Donation The National Drug Policy and  Authority Regulations, 1997 | National Drug Authority  (Uganda) |
| U7 | Guidelines on GMP Inspection of Foreign Pharmaceutical  Manufacturing Plants Use In Uganda 2007 | National Drug Authority  (Uganda) |
| U8 | Report on The Regulation of Medical Diagnostics and  Devices in Uganda | Simon Peter Rugera and Henry  Kajumbula (Uganda) |
